# Gesture enhances learning of a complex statistical concept

**DOI:** 10.1186/s41235-016-0036-1

**Published:** 2017-01-30

**Authors:** Linda Rueckert, Ruth Breckinridge Church, Andrea Avila, Theresa Trejo

**Affiliations:** Psychology Department, Northeastern Illinois University, 5500 N St Louis Ave, Chicago, IL 60625 USA

**Keywords:** Video Instruction, Spatial Concept, Iconic Gesture, Speech Content, Representational Gesture

## Abstract

Prior research has shown that gestures that co-occur with speech can improve understanding of abstract concepts by embodying the underlying meaning of those concepts, thereby making them more accessible to the listener. The present study examined the effect of gesture on undergraduate students’ learning of a complex statistical concept (analysis of variance; ANOVA). Students in three classes watched a brief video in which the speaker explained the conceptual background of ANOVA while using gesture and students in three other classes saw a similar video with the same speech, but no gesture. Students who saw the gesture learned significantly more, as measured by the increase in scores between a pre-test and a post-test. These results suggest that teachers can enhance students’ learning through the strategic use of gesture.

**Electronic supplementary material:**

The online version of this article (doi:10.1186/s41235-016-0036-1) contains supplementary material, which is available to authorized users.

## Significance

This study adds to the existing literature on the role gesture plays in learning. This study is the first to examine the effect of gesture on undergraduate students learning of a complex statistical concept (analysis of variance; ANOVA). The population of college students in this study is unique, reflecting low-income status, first generation, and ethnic diversity. Moreover, this is the first study examining the effect of gesture on learning in college students in the context of a college classroom. Previous studies have examined the effect of gesture in children’s learning, some of which examined instruction in a one-on-one setting (Cook & Goldin-Meadow, 2006; Koumoustakis et al., 2013) and some in a classroom setting (Church, Ayman-Nolley, & Mahootian, 2004; Cook, Duffy, & Fenn, 2013). Previous research examining gesture in instruction with adults have only used a one-on-one tutorial context (Carlson, Jacobs, Perry, & Breckinridge Church, 2014).

Examining how gesture enhances learning has important implications for science, technical, engineering, and mathematics (STEM) education, where the playing field is not level among individuals with few versus many economic resources (Chen, 2013). Gesture, which spontaneously occurs with speech during communication of math and science concepts, conveys concrete, embodied, and intuitive representations that compliment speech content (Alibali & Nathan, 2012; Alibali, Nathan, Fujimori, Stein, & Raudenbush, 2011; Hostetter & Alibali, 2008). Moreover, previous research on children shows that when gesture is included in instruction of math, learning is significantly more likely than when it is not (e.g. Cook et al., 2013; Koumoutsakis, Church, Alibali, Singer, & Ayman-Nolley, 2016). Thus, research on gesture in education may be able to level the playing field by making abstract and complex math and science concepts accessible (Church et al., 2004).

Moreover, online instruction was an educational innovation expected to decrease the divide between those with few and those with many economic resources. Examination of gesture effects on learning can have important ramifications for online instruction. Some online learning settings include either written or spoken language for which the speaker (and therefore his/her gestures) is not visible. In fact, when gesture’s effectiveness in instruction was compared between a video and face-to-face venue, it was found that without gesture, video instruction was wholly inadequate for mathematics learning in elementary school children compared to video instruction with gesturing (Koumoutsakis et al., 2016). The current research examines how college students who are diverse and an under-represented population respond to digital instruction with and without gesture. Thus, we see this research as contributing to our understanding of how gesture can enhance learning using a digital medium, a medium that opens access to students from diverse populations.

### Gesture enhances learning of a complex statistical concept

Educators are always eager to adopt pedagogical methods that improve their ability to convey information. One important realization is that instructional input can come in the form of many types of visual and verbal representations. How these representations are linked may be the key to making even the most abstract concepts understandable. This is particularly true in the teaching of mathematics where visual representations such as graphs have to be linked with abstract symbolic representations such as equations and verbal descriptions done by an instructor or student (Alibali et al., 2014) and particularly relevant in the efforts of STEM education where mathematical concepts need to be linked with other science disciplines (Walkington, Nathan, Wolfgram, Alibali, & Srisurichan, 2011).

A plethora of research has shown that information during communicative activities is conveyed non-verbally as well as verbally (cf., Goldin-Meadow, 2005; Kelly, Manning, & Rodak, 2008). In particular, spoken communication is often accompanied by representational gesture or gesture that conveys imagery related to accompanying speech (McNeill, 1992). Research efforts have focused on what role these gestures play in communication.

One area of research particularly relevant to this study is communication in an educational setting. Teachers and students frequently gesture when they talk about academic topics such as math and science (Crowder & Newman, 1993). As it turns out, this gesturing during teaching situations correlates with learning (Church, 1999; Church et al., 2004; Singer & Goldin-Meadow, 2005; Cook & Goldin-Meadow, 2006; Cook et al., 2013; Goldin-Meadow, Alibali & Church, 1993; Koumoutsakis et al., 2016; Novack & Goldin-Meadow, 2015). For example, gesture provided as input to the learner was significantly beneficial for elementary aged children’s learning of math concepts (Church et al., 2004; Cook et al., 2013; Koumoutsakis et al., 2016; Singer & Goldin-Meadow, 2005; Valenzeno, Alibali, & Klatzky, 2003) and science concepts (Singer, Radinsky, & Goldman, 2008). Although little research has been done on the role of gesture input for adult learning, some has shown that gesture produced during instruction benefits the learning of spatial concepts—specifically, physical causality instantiated in gear problems (Alibali, Spencer, Knox, & Kita, 2011; Carlson et al., 2014) and complex math concepts such as polynomials (Louden et al., 2015; Rueckert, Church, Saucedo, & Alibali, 2015).

The current study extends previous research on gesture’s role in instruction to young adults’ learning of a commonly taught statistical concept, ANOVA. For students who want to pursue graduate education or careers in STEM, having a solid understanding of statistics is particularly crucial. However, a recent review discovered that many students find statistics courses particularly difficult and often do not gain a deep understanding of the topic (Garfield & Ben-Zvi, 2007). In the United States, even our best students are choosing non-STEM careers over STEM careers (Bettinger, 2010) and the rate at which students choose STEM careers is much lower than other countries (Chen, 2013). This trend appears to be particularly strong for female students, students from low-income backgrounds and for students who perform poorly in college STEM classes (Chen, 2013). Thus, finding alternative methods for teaching STEM-related classes that help make abstract and seemingly opaque concepts accessible would be an important teaching innovation. We argue that using representational gesture in combination with speech during instruction is a teaching tool that appears to improve learning, especially learning of STEM-related topics such as math and science. However, the usefulness of gesture as a teaching tool has not been well explored in the college classroom. The purpose of the present study is to extend what is known about the benefits of gesture for learning to adults learning a complex statistical concept (ANOVA) in a real classroom setting.

## Method

### Participants

All participants (27 men, 67 women) were undergraduate students attending Northeastern Illinois University (NEIU), an urban college institution located in Chicago, Illinois, characterized by ethnically diverse, low-income, and first generation students. Most (n = 80) were Psychology majors who were enrolled in a required statistics and research methods course. The other 14 were enrolled in a statistics course taught in the Math department and majored in various STEM fields. Participants were distributed across the following ethnicities: 41% Latino/a, 27% White, 10% African American, 6% Asian, 16% biracial or “other.” English was reported as the primary language for 65% of the participants.

### Materials

All materials have been made available online. See the “Availability of Data and Materials” section.

#### Video instruction

Two versions of an instructional video were developed that presented a brief (about 5 min) lecture on the conceptual background of ANOVA. We chose to use a videotaped lecture instead of a face-to-face lecture because we could have greater control over a variety of confounds such as tone of voice, speech content, and facial expression.

Each video included the same PowerPoint slide show accompanied by a lecture given by a tenure-track full-time professor who has taught this class frequently. The audio portion of the lecture was embedded within the PowerPoint and was thus identical between the two conditions. To create the video, the slide show was projected on to a screen in a classroom at NEIU. The instructor stood to the left of the screen and lip-synced the audio part of the lecture, which was actually played through the projection system. In one version of the video (speech alone), the speaker stood still and did not move her arms at all but alternated between facing out to the audience and looking toward the PowerPoint slides. In the other version (speech plus gesture), the instructor produced scripted gestures to highlight and draw links between relevant information in the slides and conceptual elements of ANOVA. For example, gestures were designed to illustrate the different types of variance underlying the ANOVA formula. The gestures used were similar to those this instructor naturally uses when lecturing on this topic.

Using a fictionalized example of a study examining the effect of pain medication on headaches, one slide depicted number of headaches and means for individuals in the treatment group (those taking pain medication), those in the placebo group (taking a pill that was placebo), and those in the control group (those who took no medication; see Additional file 1). When the speaker said “It’s a measure of how much everybody’s score differs from everybody else,” she gesturally indicated “total variance” by waving her hand around all scores. When she said “The between-groups variance is a measure of how much the each group’s mean differs from the grand mean,” she gestured “between-groups variance” by pointing to the means for each group followed by the grand mean, and when she said “The within-groups variance is a measure of how much a person’s score differs from the mean for their own group,” she pointed to individual scores within a group and then the mean for the group. The lecture was designed to convey a conceptual understanding of ANOVA and did not include any actual mathematical calculations.

The appearance of the instructor (including facial expression and dress) and the time the instructor spent looking at the audience and at the slides was equated for the two conditions. Because the audio portion of one video was embedded in the PowerPoint presentation, the speech content, vocal tone, and vocal modulations were identical for both the experimental and control videotapes. The only element that differed between the two instructional videos was the presence/absence of gesture.

#### ANOVA pretest and post-test

To measure students’ understanding of ANOVA a ten-item multiple choice test was developed that covered the information presented in the video. The questions on these tests were designed to assess a conceptual understanding of ANOVA and did not include questions about mathematical calculations (see the “Availability of Data and Materials” section).

#### Demographic questions

Students were also asked to provide demographic information on ethnicity, age, and gender.

### Coding

Our primary dependent measure was the change in test scores from the pretest to the post-test (post-test number correct – pretest number correct).

### Procedure

The speech plus gesture video was shown in three classes with a total of 51 students. The speech alone video was shown in three classes with a total of 43 students. The two groups did not differ in gender, age, ethnicity, or whether English was their primary language. All testing took place during the first 20 min of the regular class time. After students signed the Informed Consent form, they were asked to answer the ten items on the ANOVA pretest and were given about 5 min to do so. Then the video was shown to the entire class. Immediately after the video was shown, students were asked to take the same ten-item test (the “post-test”). Testing was done at the time in the semester after students had learned some basic inferential statistics, including the t-test, but before they started to learn about ANOVA.

## Results

The dependent variable is the change in score between the pre- and post-test (post-test raw score minus pretest raw score). An independent t-test showed that the change score was significantly greater for students in the speech plus gesture condition (*M* = 2.20, *sd* = 2.14) than for those in the speech alone condition (*M* = 0.86, *sd* = 2.51), *t*(92) = 2.78, *p* = 0.007, *d* = 0.57. Although the degree of change in the speech alone condition was very small, a one-sample t-test showed that it was significantly greater than 0, *t*(42) = 2.25, *p* = 0.03. Thus, both versions of the video resulted in significant learning, but the amount of learning was greater when gesture was included.

A multiple regression analysis with change score as the dependent variable and condition (speech alone or speech plus gesture), gender, age, ethnicity, and primary language as independent variables showed a significant effect only for condition, β = 0.28, *t*(82) = 2.61, *p* = 0.01.

A comparison of the change scores for different classes within the same condition showed that there were no significant differences between classes, (speech alone: *F*(2,42) = 0.29, *p* = 0.746, speech plus gesture: *F*(2,48) = 1.05, *p* = 0.357).

## Discussion

We found that gesture significantly enhances undergraduates’ learning of a complex statistical concept compared to speech alone. This result extends previous reports supporting the benefits of gesture for learning in children (e.g. Church et al., 2004; Cook et al., 2013; Koumoutsakis et al., 2016; Singer & Goldin-Meadow, 2005) to adults. It should be pointed out that what was conveyed in the videotaped lecture and what was assessed on the tests was not a method for calculating statistics, but rather a conceptual basis of ANOVA (e.g. the difference between between-groups and within-groups variance). This type of abstract conceptual information may be especially likely to benefit from embodiment through instructional input provided in complimentary gesture. The gesture serves to make the abstract concepts more concrete and accessible to students in ways that have been shown in the literature such as illustrating words through imagery (Singer et al., 2008), establishing links among relevant contextual references for the learner (Alibali & Nathan, 2012) and drawing attention to aspects of content in speech (Alibali & Nathan, 2012).

In this particular study, we used gestures that naturally occurred with the teacher’s ANOVA lecture. These gestures matched the speech and were honed and scripted to provide a spatial link between speech information and the information contained on the slides about the concepts of within, between, and total variance of data.

There are a few interesting facts about co-speech gestures (gestures that occur with speech) that make them particularly interesting as vehicles for teaching. First, they are frequently produced during communication (Goldin-Meadow, 2005; Kelly et al., 2008; McNeill, 2000), yet until recently, gestures have been less systematically studied than, for example, speech, as part of communication input. Second, the frequency of gesture production does not correlate with age or with particular types of discourse. Gestures occur frequently across the lifespan suggesting that gesture’s function may not be tied to some language or cognitive capacity associated with a particular developmental stage or age group (McNeill, 1992). In addition, the production of gesture occurs in educational contexts (Alibali & Nathan, 2012; Crowder & Newman, 1993; Singer et al., 2008). It is certainly the case that teachers gesture when they teach spatial concepts like math concepts (Alibali et al., 2013 Alibali & Nathan, 2012; Alibali, Nathan, et al., 2011) and students gesture when they talk about math and science concepts (Church & Goldin-Meadow, 1986; Singer et al., 2008). Fourth, co-speech gestures convey information in the form of iconic imagery tied to the content of the accompanying speech (McNeill, 2000). This imagery depicts information using non-verbal representations rather than words. Gesture can provide a complimentary image of the content conveyed in speech such as when a person makes a large, circular gesture while saying, “It was a big, round one” or a gesture that depicts throwing a football while saying, “Let’s throw the football.” This feature of gesture has particular relevance to instruction of spatial concepts such as those found in science and mathematics when often the topics are not visible. Research that examines gestures in math and science classrooms find that explanations of concepts are often accompanied by rich gestural representations that can ground even the most abstract terminology (Crowder & Newman, 1993; Marghetis & Nunez, 2013; McNeill, 1992; Singer et al., 2008). It has been suggested that mathematics is cognitively constructed through our sensory and motor experiences (Lakeoff & Nunez, 2000), which makes representational gesture a particularly advantageous vehicle for explaining mathematics. This study further demonstrates that the gestures that occur in the classroom may serve a significant function for learning.

### Limitations

We think that this study is a very important introductory step towards understanding the role gesture might play in how classroom instruction, and in particular, video-streamed instruction, can improve learning of STEM related concepts. There are some limitations, however, to consider. First, our sample, although diverse and representative of the types of students that STEM education has been designed to help, was still limited. Students were mostly urban psychology students and women, with only a small representation of math students majoring in other STEM disciplines, and men. Future work needs to expand to a variety of other college populations including men, traditional college students, and those in rural settings. Second, we acknowledge that using a scripted video of a lecture is different from a face-to-face natural lecture context where discussion between the teacher and students might be more prevalent. Capturing the effects of gesture in a natural situation is extremely difficult for making causal inferences; there was a necessary external/internal validity trade-off. However, the fact that the video was presented in an actual statistics class rather than in a lab room enhances the external validity. Third, this study was done with just one faculty member—although held constant across speech only and speech and gesture instruction, the instructor may have had her own unique contribution to learning that could have either diluted or enhanced the effects of gesture. Future work should include a number of different teachers to test the generalizability of gesture’s influence on learning. Fourth, it is possible that the students could have noticed the lip-syncing and may have been distracted by it. However, for most of the lecture the speaker’s face was either not visible due to the fact that she was looking at the slide, or it was partially shadowed, making her lip movement difficult to see.

Finally, this study did not determine exactly what role gesture played for learning. There are a number of roles gesture could have played other than conveying abstract information through embodied or concrete imagery. One role would be as a way to link multiple representations such as those conveyed in speech or those conveyed on the slides. This role of linking representations has been noted in previous research (Alibali & Nathan, 2012). Another role gesture could have played is to draw attention to the lecture simply through compelling movement. That is, the content in gesture may not have been helpful but the gestural movement may have kept viewers alert and attentive to the lecture. However, previous research which included other conditions to pull apart gesture’s role in attention (using yellow highlighting on a white board while the instructor spoke; Bem et al. 2012) or using non-imagistic gestures with speech (Koumoustakis et al., 2013) showed that speech with gesture was significantly better for learning than highlighting or gesture movement with no meaning.

One other possible role that gesture could have played is that gesturing conveyed to the viewer that the instructor is a likable person. Being more likable as an instructor may make students learn more.

We followed a well developed design, modeling other research examining the effect of gesture in instruction for children; comparing speech only instruction versus speech and gesture instruction (e.g. Church et al., 2004; Cook et al., 2013; Cook & Goldin-Meadow, 2006; Koumoutsakis et al., 2016). This study expands this methodology to examine adult learning and reflects an initial but important step in understanding whether the presence of gesture impacts learning. Future work, of course, is needed to get at the specific role gesture plays in communication.

It is our contention that the gestures used in the instruction for ANOVA were representational in nature using iconic gestures (indexical points and illustrating gestures) to convey within- and between-groups variance by linking spatial information on the slide with accompanying speech. Thus, the likely role gesture played in this research was to link relevant representations of ANOVA (in the form of concrete examples, equations, and speech) as well as to encapsulate the basic notion of variance using spatial imagery (circling around the means of all the experimental groups to relay the notion of between-groups variance) as these types of gestures were used predominantly throughout the lecture. So it is not surprising that students showed improvement when tested on aspects of variance after watching the speech plus gesture video.

The results of this study have a number of important ramifications. The online instructional venue has increased dramatically over the course of the last decade. Many online courses include a speaker whose entire body is visible and therefore presumably include gesture. However, others involve only written materials or a voice-over (e.g. Khan Academy). Given the importance of gesture for learning abstract concepts, future designers of web-based courses should seriously consider including a speaker who gestures. Furthermore, even when a speaker is present, either online or in a live classroom, they should consciously monitor their own gesture to ensure they are maximizing its potential. Whether or not alternative methods of indicating important information, such as movement of a pointer or cursor, can have benefits similar to gesture is an open question.

To date, gesture has been shown to improve children’s and adults’ understanding of a number of abstract spatial concepts such as those taught in math and science classes. Embodiment through the use of gesture has great potential to help students achieve a deeper understanding of many abstract scientific and mathematical concepts. Our study suggests that perhaps one way to increase student’s achievement in STEM-related courses and, in turn, STEM careers is to introduce representational and embodied gesture as a compliment to speech instruction.

## Additional files

Additional file 1: PowerPoint slides and script. (DOCX 225 kb)

Additional file 2: De-identified raw data. (XLSX 13 kb)

## Abbreviations

ANOVA: analysis of variance
NEIU: Northeastern Illinois University
STEM: Science, technology, engineering and mathematics
