# Supplementary material for: Gesture enhances learning of a complex statistical concept
Source: Cogn Res Princ Implic. 2017 Jan 30;2:2. doi: 10.1186/s41235-016-0036-1 (PMC5281660; doi:10.1186/s41235-016-0036-1)
Supplement: Supplementary file 1 — PowerPoint slides and script. (DOCX 225 kb) [file 41235_2016_36_MOESM1_ESM.docx]

Appendix

Powerpoint slides and script

(to see the full Powerpoint and the actual video that was shown, go to http://homepages.neiu.edu/~lruecker/anova_study)

**Slide #1**.


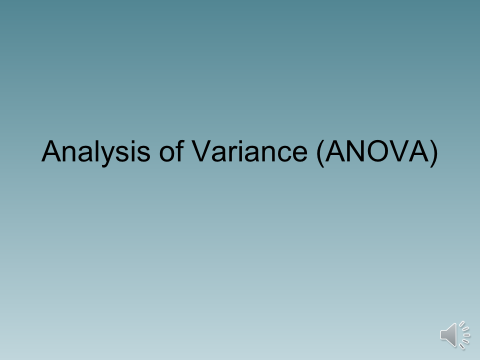


Okay, today we’re going to start talking about analysis of variance, which is also known as ANOVA.

**Slide #2**


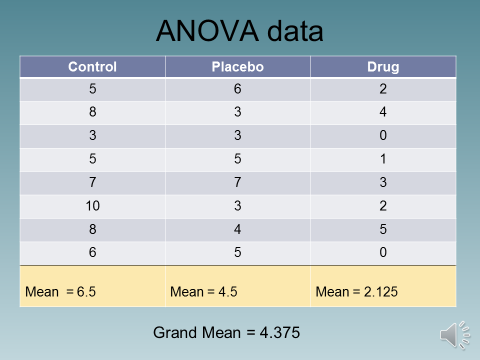


This is some data we are going to use as our example. It’s for a study on the effect of a drug (gesture a *pill* using thumb and forefinger indicate the length of a pill) on the number of migraine headaches a person gets (point to head). So the dependent variable is the number of headaches (point to head) a person got in one month and the independent variable is the drug (gestures *pill*). There are three groups: (point to each group). The control group doesn’t take anything (hand wave indicating *there is nothing*), the placebo group does take a pill (gestures *pill*) but it doesn’t have anything in it (hand wave indicating *there is nothing*). The drug group takes a pill that has the drug in it (gesture pill). The mean number of headaches for the control group was 6.5 (point to mean for the control group), the placebo group had 4.5 headaches (point to mean of the placebo group) and the drug group had 2.125 headaches (point to mean of the experimental group). The grand mean, which is the mean for everybody combined, is 4.375 (indicated all scores then point to grant mean).

**Slide #3**


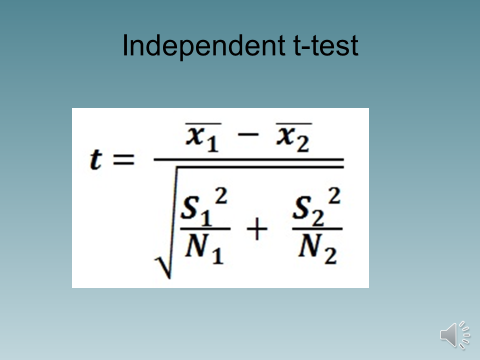


You’ll recall the formula for the independent t-test, which is used to compare means of two groups. The numerator, or top part, is one mean minus the other (point to numerator). The denominator or bottom part is the variance of the two groups (point to denominator). Uh, but now we have more than two groups, so we can’t just subtract one mean from the other.

**Slide #4**


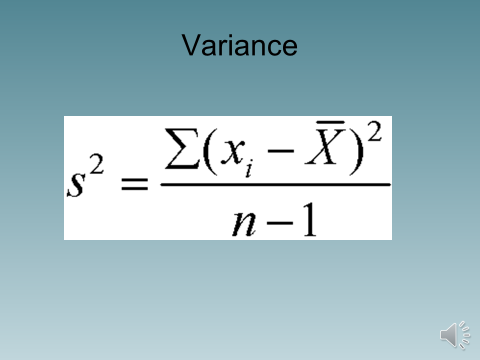


Recall the formula for the variance. Uh the variance measures how much, how much different people differ from each other and it’s calculated by taking each person’s score and subtracting the mean (point to formula).

**Slide #5**


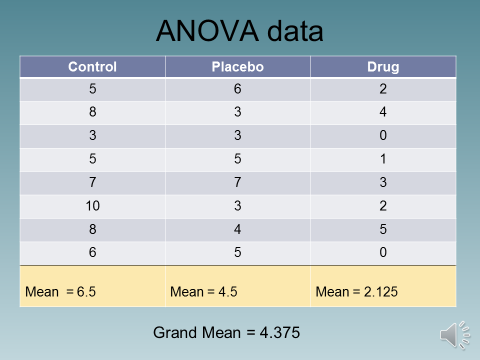


We’re now going to differentiate between different types of variance. The variance that you’re already familiar with is the total variance. It’s a measure of how much each person’s score differs from the grand mean (point to individual scores, then grand mean). It’s a measure of how much everybody’s score differs from everybody else (gesture around all data).

We’re going to break that down in to two (gesture 2 fingers) different parts. The within-groups variance is a measure of how much a person’s score differs from from the mean for their own group (point to two individual scores followed by group mean). So it’s a measure of how much the people within a group differ from each other (gesture around all the scores in the control group). The between-groups variance is a measure of how much the each group’s mean differs from the grand mean (point to two group means followed by a point to he grand mean). So it’s a measure of how much the means for the groups differ from each other (gesture that circles around all group means)

**Slide #6**


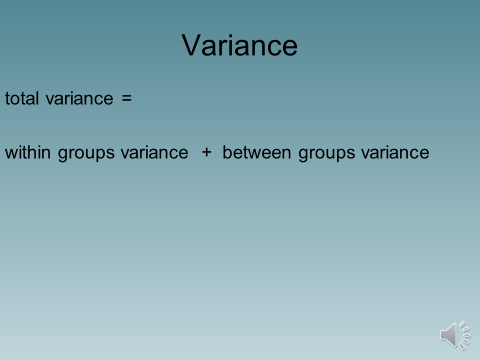


So the total variance (point to “total variance”) is equal to the within-groups variance (point to “within groups variance”) plus the between groups variance (point to “between groups variance”).

**Slide #7**


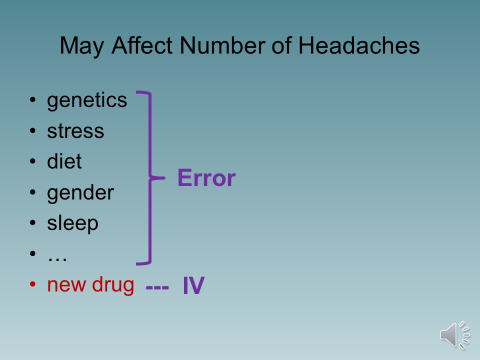


There are many things that affect the number of headaches (point to head) somebody gets. Um, it could be affected by their genetics, by stress, by diet, gender, how much sleep they got. In fact there is an infinite number of things that can affect the number of headaches somebody gets (point to each item listed on the powerpoint; e.g. “stress,” “diet”, ect.). One thing that might affect the number of headaches is the drug, (point to “drug” on the list of items that could affect occurrence of headaches) which is our independent variable . Uh, but it can also be affected by any of these other extraneous variables (indicate all items on the list of factors affecting headaches). Any variable other than the independent variable we’re going to refer to as error.

So when we observe that the drug group had fewer headaches than the other groups it might be due to the drug, or independent variable (point to the ppt where “drug” is listed); but it might be due to something else, which we’re calling error (point to all other factors affecting headaches on ppt). Uh for example, maybe a couple people in the placebo group happen to be under a lot of stress and that’s what raised their mean.

**Slide #8**

**
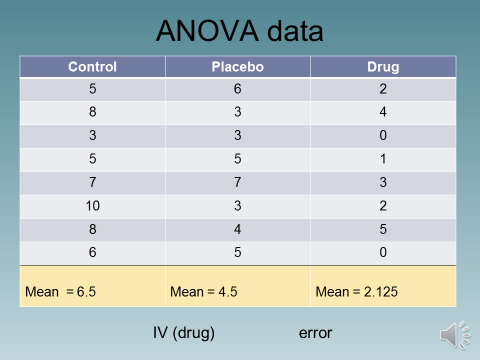
**

So now recall that the between groups variance is a measure of how much the different groups differ from each other (indicate means for each group). That could be affected by the IV or drug (point to “drug” listed on ppt), but it could also be affected by error (point to “error”). The within groups variance, on the other hand, is a measure of how much people differ from the other people within their own group (indicate people within one group). Within a group everybody got the same level of the IV (point to drug label at top of column), so the IV can not affect any differences. The within groups variance is only affected by error (point to “error”).

**Slide #**9


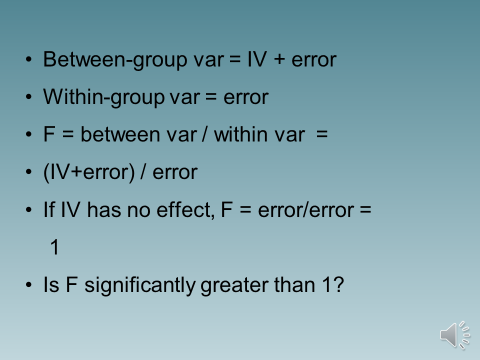


So we’re going to calculate the between-groups variance (point to “between groups variance”) which is equal to the IV plus error, and the within-groups variance ( point to “within groups variance”), which is only equal to error. We’re then going to calculate F, which is the between groups variance divided by the within groups variance (point to “between groups variance divided by within groups variance”). So that is equivalent to IV effect plus error (point to correspondence with previous equation) divided by error (point to correspondence with previous equation). Uh, now if the IV has no effect, this will be 0 (cover up IV in previous equation). So F will be simply error divided by error (point), and as we all know, any number divided by itself is one (point to “1”). But if the IV does have an effect an effect (point back to previous equation), F will be greater than one.

So the question is, is F significantly greater than one?
